# Morpho-Anatomy and Mathematical Modelling in *Lilium philippinense* Baker from Cordillera Central Range, Philippines

**DOI:** 10.21315/tlsr2023.34.3.12

**Published:** 2023-09-30

**Authors:** Jennifer C. Paltiyan-Bugtong, Rey G. Lumpio, Jones T. Napaldet

**Affiliations:** 1 Biology Department, Benguet State University, 3rd Floor CNS Building, Km. 5 La Trinidad 2601, Benguet, Philippines; 2 Climate Smart Agriculture Center, Benguet State University, 3rd Floor RnE Building, Km.6, La Trinidad 2601, Benguet, Philippines

**Keywords:** Monocot Plant with Dicot Characters, *Lilium philippinense*, *Lilium formosanum*, Correlation, Regression

## Abstract

The study presents the morphology, anatomy and mathematical modelling in Benguet lily (*Lilium philippinense Baker*), a threatened species from the Cordillera Central Range and was often misidentified with the weedy *L. formosanum*. The plant is an annual herb with linear, spiral leaves; pure white, perfect, funnel-shaped, showy flowers; septicidal elongated capsule; and, brown, light, winged seeds. New findings in the study include the description of the capsule and seeds, biometric measurements of the different plant organs, the significant correlation and regression model of plant height and stem diameter for certain floral measurement, and its diagnostic characteristics vis-à-vis *L. formosanum*. Interesting findings on the taxon’s anatomy show a cross between a typical monocot and a typical dicot anatomy. It has a bifacial leaf structure (a common dicot character) but its spongy layer is not as widely spaced like in dicot leaf. The stem has a distinct cortex and pith (a dicot character) but has a scattered vascular bundles (a monocot character). Lastly, some roots have a narrow pith at the centre of the stele (a monocot character) while some roots have metaxylem elements at the centermost structure (a dicot character). Further studies need to be conducted to determine the ecological significance of these features.

**Highlights**

- In Cordillera Central Range, Northern Philippines, *Lilium philippinense* is distinct from *L. formusanum* with its pure white flowers, generally greenish stem (except some individuals having random purplish colouration) and being found only in stony or gravelly soils and never in red oxide soils.
- Significant correlation of plant size and floral measurements in *L. philippinense* could be the first report on how vegetative traits (specifically plant height and stem diameter) dictate floral characters in plants.
- Interesting anatomy of *L. philippinense* shows a cross between a typical monocot stem and a typical dicot leaf and root anatomy.

## INTRODUCTION

The genus *Lilium* are perennial herbs of great commercial significance due to their unsurpassed beauty. Their significance was recognised as early as in the ancient civilisations. For example, *Lilium candidum* (Madonna lily) was used as a cut flower in the middle Minoan IIIA–B period (ca. 1750–1675 BC) and known to be of biblical importance. In China, lilies have been cultivated for food and medicine for at least 2,000 years (Haw 1986). Today, lilies are one of the most important cut flowers and pot plants of the worldwide horticultural bulb trade. Lily species and hybrids are also used as garden plants. Due to their importance, the natural species of *Lilium* were highly hybridised and selectively bred. According to Flora of the World (http://www.plantsoftheworldonline.org/), 118 natural species of *Lilium* are recognised but more than 300 cultivars are registered per year and the accumulated number of registered lily cultivars is more than 9,400 (http://www.lilyregister.org). This shows that majority of the studies on *Lilium* are on its hybridisation, cultivation, pest or disease management and some on its phylogenetic treatment. However, studies on the ecology of the natural populations in the wild were not given equal emphasis. Such was the case of *Lilium* species in the Cordillera Central Range (CCR), Northern Philippines. Three species, namely *L. philippinense*, *L. formosanum* and *L. longiflorum*, were reported by Balangcod *et al*. (2011) to naturally occur in CCR but according to Co’s Digital Flora (Pelser *et al*. 2011–), only *L. philippinense* occurs naturally in CCR. The latter report may have lead Napaldet (2017) to misidentify and describe *L. formosanum* as *L. philippinense*. He presented the morphological and anatomical characterisation of *L. philippinense* in the CCR but more careful analyses showed that the plant samples were actually of *L. formosanum*. Recent surveys also noted that *L. formosanum* occurs in several sites in CCR but its mode of introduction is not yet fully understood. Currently, *L. formosanum* does not appear to be displacing *L. philippinense* as they were observed to occur in different sites. This highlighted the need to update and thoroughly characterise the *Lilium* species in the CCR as an important field guide and for better understanding of the wild populations; hence, this study.

Locally, the study is part of the on-going effort to properly characterise the many indigenous and endemic plants in the CCR. This area is one of the 15 biogeographic zones in the Philippines (Ong *et al*. 2002) and is both culturally and biologically important. It is the home of several groups of indigenous people and it harbours a unique set of floral species not found in other parts of the country (Balangcod *et al*. 2011; Jacobs 1972; Merrill & Merrit 1910). However, it has become evident in recent years that studies on plant diversity in the region has declined and have received less support from concerned agencies perhaps due to the lack of appreciation for taxonomy and biodiversity at the local level. Likewise, the acculturation of the local communities and the diminishing emphasis on their indigenous knowledge system (IKS) towards environment protection have led to habitat degradation as they integrate into a cash-crop economy. Navarro and Saldo (2000) noted the intensifying pressures that drive species extinction in the area such as forest conversion, mining activities, pollution and hunting. With these, baseline studies on the biodiversity of the CCR are imperative towards conservation.

The study characterised the morpho-anatomical characters of *L. philippinense* to clarify its identity from related species. Thi Tran *et al*. (2022) emphasised the importance of morpho-anatomical studies as still relevant even today in establishing or clarifying the systematics of plants. The study was also able to document a strong correlation and mathematical models between the vegetative and floral characters in *L. philippinense*. This is an interesting addition to mathematical botany. The mathematical precision in plants was long been recognised including the discovery of Fibonacci sequence, the tree architectural models (Hallé *et al*. 1978)) and the several mathematical model in plant physiology (e.g., Nyman & Brown 1996) and in molecular botany (Ledford 2013). This study could be the first report on the mathematical model on how vegetative traits (specifically plant height and stem diameter) dictate floral characters in plants as no other studies were cited in available literatures.

## MATERIALS AND METHODS

### The Subject Plants

*L. philippinense* was first described by John Gilbert Baker in the journal Garden Chronicles in 1873. It is closely allied to *L. longiflorum* but is distinct by having narrower funnel-shaped flower. It was long thought to be endemic in the CCR, Northern Philippines (Balangcod *et al*. 2011) but plant taxonomic websites (e.g., Co’s Digital Flora, World Flora Online, Plants of the World Online) listed it recently as indigenous both in the Philippines and Taiwan. Recent studies specific on this taxon include the study of Balangcod *et al*. (2011) on its geographical distribution and by Alipio-Ayban *et al*. (2019) on its phenology.

On the other hand, *L. formosanum* is a native of Taiwan but was widely introduced as bulb flower and had escaped cultivation. It was even listed as a weed in several countries (Warner *et al*. 2006) which is a testament to its hardiness. It is distinct from other related lilies by its funnelform white flowers with purple tinge, linear leaf, presence of papillose nectaries, and leaf axils are without bulblets. In the Philippines, Co’s Digital Flora reported it to occur only in Batanes but recently was cited in several sites in the CCR.

### Sampling and Morpho-Anatomical Characterisation

**Figure 1:**
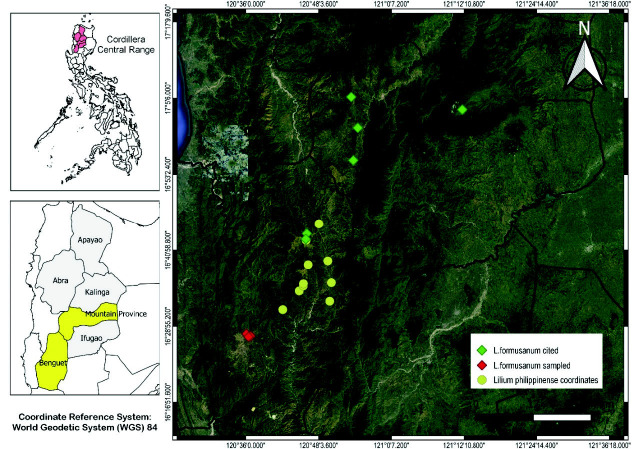
Map of Benguet in Cordillera Central Range showing the populations of *L. philippinense* and *L. formosanum* sampled in the study. (Created using QGIS)

We sampled 10 populations of *L. philippinense* along Halsema Highway in Benguet province and four populations of *L. formosanum* along the slope of Mt. Yangbew and Mt. Kalugong in La Trinidad, Benguet (Fig. 1). In every population, five individuals were randomly sampled for morphometric measurements with emphasis to include individuals with extreme characters. We limited the sampling due to the vulnerable status of the plant. Simultaneously, morphological description was also conducted. Moreover, the anatomical features of for *L. philippinense* were characterised in this study since the anatomical features of *L. formosanum* was already presented in Napaldet (2017). Plant sections of the leaf, stem and roots were prepared and described under the light microscope. Morphological and anatomical description of the plant follows Haupt (1953), Fahn (1982), Bell (1991; 2008) and Shipunov (2018).

The stomata in the leaf were also observed. Stomatal imprints were derived by applying nail polish on the leaf epidermis, particularly on the abaxial epidermis. The nail polish was allowed to dry then was transferred to a clear tape then to the glass slide. The apex, base and middle part of the lamina were randomly sampled to get an adequate representation. These imprints were used to compute for the stomatal density and index. Stomatal density was computed by counting the number of stomata per square millimeter. The area was determined through ImageJ, a free software. Using the same area and program, the stomatal index was determined by dividing the number of stomata by the total number of epidermal cells:


$$=\text{I}=\frac{\text{S}}{\text{E}+\text{S}}\times 100\%$$

Where I = stomatal index, S = number of stomata per unit area and E = total number of epidermal cells.

Lastly, general observation on the populations’ habitat were assessed particularly on the soil and slope conditions.

### Statistical Analyses

Descriptive statistics such as mean, range and standard deviation were used to present and analyse the morphometrics of the two plants. Since there are multiple populations sampled for *L. philippinense*, we employed ANOVA to determine significant differences between populations. This determines if the taxon is polytypic or homotypic. Lastly, we correlated the plant height and stem diameter with the floral measurements and developed regression models for prediction purposes. This is an interesting addition to mathematical botany and could be the first report on how vegetative traits (specifically plant height and stem diameter) dictate floral characters in plants.

## RESULTS

### Morphological Description and Morphometrics of *L. philippinense* Baker

**Figure 2:**
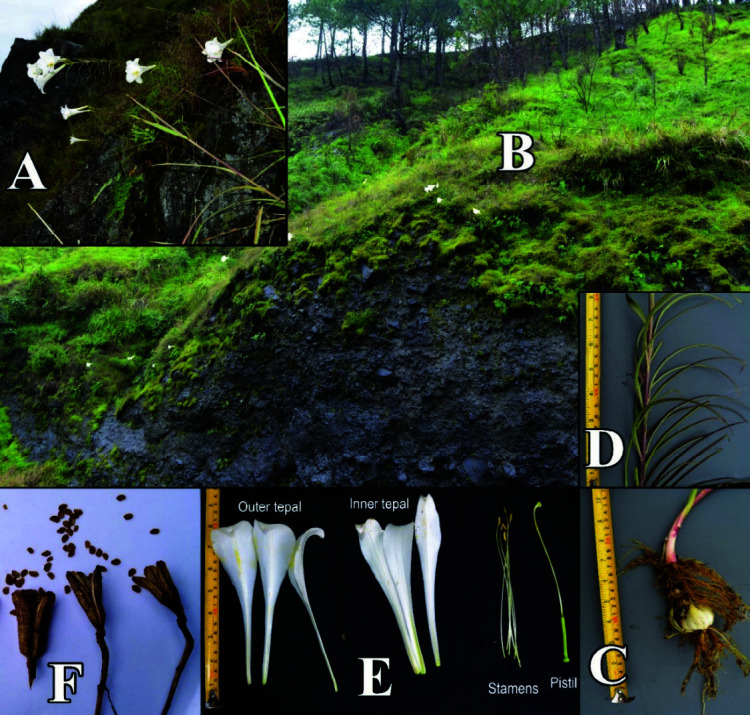
(A, B) *L. philippinense* Baker in its natural habitat, (C) its root system, (D) the stem and leaves, (E) the floral parts, and (F) the fruits and seeds.

The plant is an annual herb arising from a white bulb with fibrous roots arising from the subterranean base part of the stem and contractile roots below the bulb. It grows at the start of the rainy season (June) and dies at the end of the rainy season (October). It is usually less than 1 m tall with greenish stem. In individuals exposed to full sunlight, the stem may turn purplish in random manner. The leaves are spiral, sessile, linear, mid-green, glabrous, entire, 7.50 cm–1.80 cm long × 0.30 cm–0.70 cm wide with the middle leaves being the longest while basal and apical leaves tend to be shorter, apex acute, venation parallel with only the midrib being prominent. Flowers are generally solitary with some individuals having two or three flowers, terminal, pedicillate, showy, salviform. Tepals pure white with the mid-vein somewhat greenish particularly at the base, oblanceolate, 2-whorled arranged imbricate-alternate; three outer tepals, generally longer but narrower (13.50 cm–24.50 cm long × 2.10 cm–4.90 cm wide) with more revolute apices; three inner tepals, generally shorter but broader (12.90 cm × 23.80 cm long × 3.45 cm × 7.20 cm wide) with less revolute apices. Stamen 6, filamentous, inserted, apostemonous; filament white, 9.30 cm–18.30 cm long; anther dithecal, subbasifixed, 0.50 cm–1.80 cm long × 0.13 cm–0.55 cm wide; pollen grains yellow, globose. Gynoecioum syncarpous, 3; ovary elongated, superior with axile placentation, 2.00 cm–4.70 cm long at 0.30 cm–0.60 cm diameter, green, trilobed; style terminal, 9.00 cm–17.00 cm long; stigma trilobed, 0.50 cm–1.20 cm diameter with sticky surface. Fruit is a septicidal capsule that opens basipetally, 3.90 cm–6.10 cm long at 1.30 cm–2.20 cm diameter. Seeds are several, brown, light and flattened (obvious for wind dispersal), oblong, 0.40 cm–0.60 cm long × 0.55 cm–0.85 cm wide. Refer to Fig. 2 for the morphology of the plant and Table 1 for the morphometrics.

Flowering usually starts from July to September then the leaves start to die with the corresponding development of the ovary into the fruit until its opening from October to November. The spectacular flowering of the plant is usually not appreciated by the general public because of heavy rain during these times that cause damages to the community and thus the locals are busy fixing stuff rather than appreciating the beauty within their midst. This time is also a lean season for tourism in the region.

Different populations of *L. philippinense* were sampled in the study to determine the extent of its plasticity. The morphological descriptions were consistent between the different populations but the vegetative and several reproductive measurements were significantly variable (Table 2). Plant height, stem diameter and leaf length significantly varies between populations and these are expected because vegetative characters are generally plastic. It was observed during the field data gathering that taller individuals with thicker diameter and larger leaves occur in richer but still gravelly/sandy soils compared to shorter population with thinner diameter and smaller leaves in poorer soils. However, it is interesting to note that reproductive measurements such as outer tepal length, inner tepal length, style length and ovary length were also significantly variable between populations. We observed in the field that generally, the taller the plant, the longer are their floral measurements. This clearly shows that the optimum nutrient uptake from the preferred habitat affects the overall growth of this plant. Further, this gave us the idea to look into the correlation and regression between the plant height and stem diameter with floral measurements.

### Mathematical Modelling in *L. philippinense* Baker

Table 3 presents the correlation of plant height and stem diameter with the leaf and floral measurements. Plant height was significantly correlated with leaf length, outer tepal length, inner tepal length, style length and ovary length. This verifies our field observations that plant height significantly correlates with leaf and floral length measurements. Generally, taller individuals will have significantly longer floral measurements. On the other hand, stem diameter is significantly correlated with the number of flower per individual and with anther length. It was observed that only individuals with greater diameter produce more than one flower. With these highly significant correlations, we explore regression modelling to determine how strong plant height and stem diameter can be predictors for the leaf and floral measurements.

Different regression types such as linear, power, polynomial, logarithmic and exponential were explored between plant height and stem diameter with the leaf and floral measurements; thus, several models were generated. To check how strong plant height and stem diameter be predictors for the leaf and floral measurements, the *r*^2^ values were computed. The coefficient of determination (*r*^2^) is considered as a test of accuracy for regression models with higher *r*^2^ values indicating higher accuracy of the model (Kahane 2001). Only those with better *r*^2^ values were presented (Table 4). Plant height emerged as the predictor for leaf length, outer tepal length, outer tepal width, inner tepal length and style length with moderate accuracy (*r*^2^ = 0.36–0.49). On the other hand, stem diameter is a moderate predictor for the number of flower per individual and inner tepal width (*r*^2^ = 0.38–0.44).

### Anatomical Descriptions

#### The leaf

**Figure 3:**
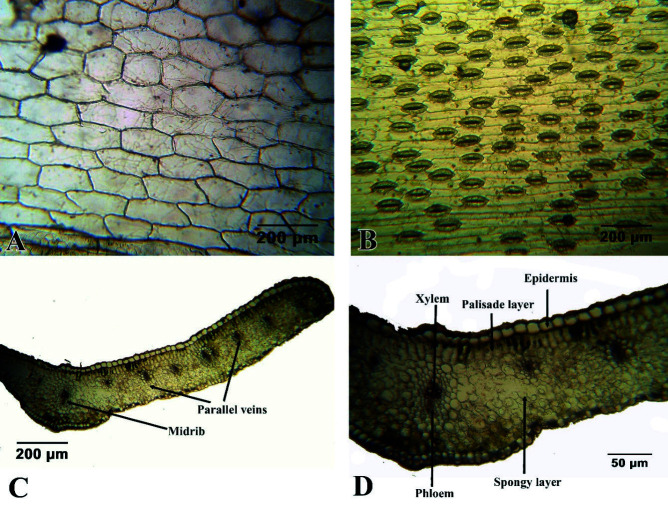
Leaf anatomy of *L. philippinense* Baker showing the (A) upper epidermis, (B) lower epidermis and the leaf layers at (C) Low Power Objective (LPO) and (D) High Power Objective (HPO).

The epidermal cells of both upper and lower epidermises are generally rectangular and slightly tapering (Fig. 3A, unstained) with slanted anticlinal walls. The periclinal wall is generally straight but some abaxial epidermal cells are curved particularly those located in between stomata (Fig. 3B, unstained). The stomata of *L. philippinense* are of anomocytic type, where stomata are surrounded by limited number of subsidiary cells which are quite alike the remaining cells. The stomata are found only in the abaxial epidermis.

The cross-sections of the leaf is shown in Figs. 3C–3D wherein the upper and lower epidermises enclose the mesophyll and regularly spaced vascular bundles. The upper and lower epidermises are both uniseriate and composed of transparent cells. The upper epidermal cells are generally larger with thicker cuticle. The mesophyll is divided into palisade and spongy layer. This mesophyll structure is quite different from the common monocot leaves which have undivided mesophyll. The palisade layer resembles a brick-like layer of rectangular cells, not elongated, which make it distinct from the spongy layer that is composed of generally larger cuboidal cells that are seemingly loosely arranged with air spaces in between cells. However, the air spaces of the spongy layer are not as prominent as those observed in typical dicot leaf. The vascular tissues are regularly spaced as consequence of the parallel venation with xylem at the upper portion and the phloem beneath.

#### The stem

**Figure 4:**
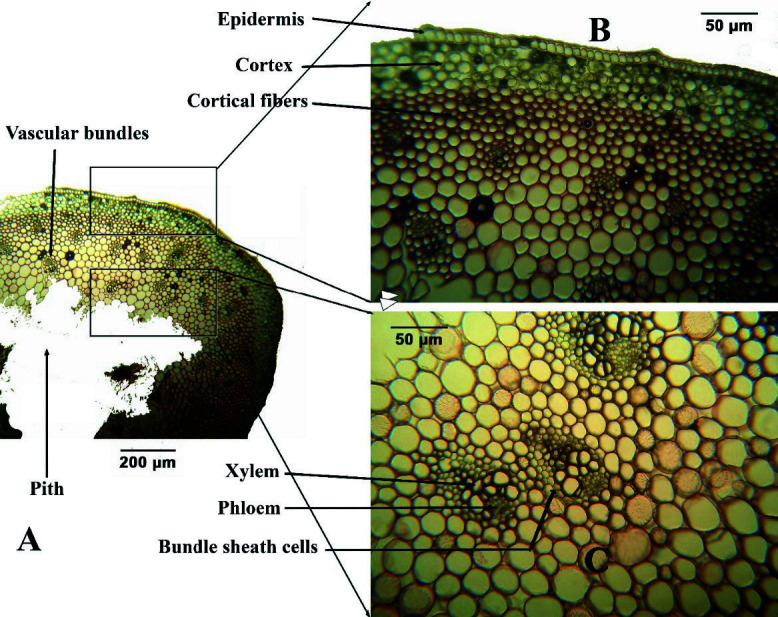
Stem anatomy of *L. philippinense* Baker at (A) LPO; at (B) HPO showing the outer tissues; (C) HPO showing the vascular bundles.

Fig. 4 shows the cross-sections of the stem that exhibits a cross between siphonostele and atactostele arrangement of vascular bundles. The single-layer epidermis covered by cuticle forms the outermost covering, followed by a cortex of 4–7 layers of parenchymtous or collenchymatous tissues and then by layers of cortical fibres. On young (apical) stem, the cortex are predominantly parenchymatous but on mature (middle) stem, collenchymatous layers developed then became schlenchymatic on older (basal) stem). After the cortical ffibres are the vascular bundles which are more concentrated at the periphery then the pith at the center which is predominantly parenchymatous. The pith has very few or no vascular bundle at all. These anatomical features of *L. philippinense* superficially resemble a dicot stem with the presence of distinct cortex that is separated from the central pith by the cortical fibres and vascular bundles forming a cylindrical-like pattern. However, the vascular bundles are arranged in a scattered manner (atactostele), albeit at the periphery, which is common arrangement in monocot stem. This leads the author to describe it as a ‘pseudosiphonostele’ arrangement.

#### The roots

**Figure 5:**
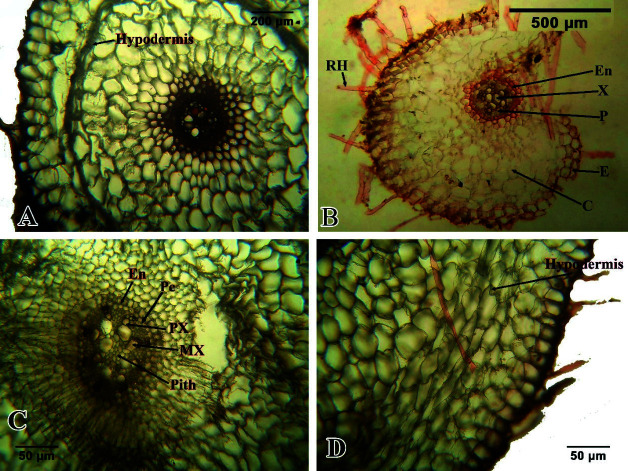
Root anatomy of *L. philippinense* Baker showing the cross-section of the contractile roots (A, C, D) and the fibrous roots (B).(Notes: C = cortex, E = epidermis, En = endodermis, MX = metaxylem, P = phloem, Pe = pericycle, PX = protoxylem, RH = root hairs).

As mentioned above, the plants have adventitious roots and contractile roots. Cross sections of adventitious roots showed a typical monocotyledon roots with few exemptions (Fig. 5B). The epidermis, consisting of single layer of rectangular cells, forms the outermost covering followed by a wide cortex then the vascular cylinder. Unicellular roots hairs were also observed arising from the epidermis of varying height. The cortex consists of generally circular, large parenchyma cells. After the cortex lies the endodermis, composed of a single layer of well-shaped cells lined with Casparian strip. This is followed by the pericycle which is composed of thin, single layer of cells. There was no well-defined parenchymatic core or pith in the vascular cylinder, instead, the center is dominated by metaxylem elements. The xylem is exarch with 5 arms and the phloem is located in between these xylem arms. This vascular arrangement resembles more of typical dicot root than a monocot one.

On the other hand, the contractile roots are larger than the annual adventitious roots and are perennial as the bulb (Figs. 5A, 5C, 5D). Its cross-sections exhibit a generally similar structure like that of the adventitious roots with few distinctions. The cortex has a suberized layer forming a hypodermis which is just few layers below the epidermis (Fig. 4A). This suberized layer protects the cortex from dehydration during dry season and is more defined in plants growing in shallow soil found on rock crevices common on the mountain’s summit. Some cells in the cortex are irregularly shaped with contorted cell walls particularly along the periphery while the rest are rectangular to hexagonal. In contrast with adventitious roots, the cortical roots have a narrow pith in the middle of the vascular bundle.

### Comparison with *Lilium formosanum* A. Wallace

The morphology of *L. philippinense* is much similar with *L. formosanum* which would explain why one could easily mistake one from the other. Both have linear leaves and showy white flowers. With regards to morphometrics, *L. formosanum* is more variable in terms of plant height, stem diameter, leaf sizes and number of flowers per individual. *L. formosanum* could grow to a much greater height and have much greater flower than *L. philippinense* (see Table 4). Additionally, *L. philippinense* is distinct with its pure white flowers, greenish stem with some individuals having random purplish colouration and being found only in stony or gravelly soils and never in red oxide soils. On the other hand, *L. formosanum* has white flowers with consistent purplish colouration along the tepal veins, purplish colouration in the stem are in checkered manner wherein purplish blocks alternate with greenish colouration of the stem and they occur in different soil types.

## DISCUSSIONS

### Morpho-Anatomy of *L. philippinense*

From a conservation perspective, the understanding of the many aspects of plant biology including the ecological and physiological ends of the spectrum are of vital importance. An appreciation of morphology and anatomy is fundamental in understanding these. Plant anatomy, along with “whole plant” physiology and comparative plant morphology, has long formed the core of general botany courses. This is the subject matter at the heart of the study of plants at the organismal level. Aside from being a source of characters for taxonomic classification and phylogeny reconstruction, plant morphology and anatomy elucidate the relationship of these structures to their functions or physiology (Pancho & Gruezo 2012), thus, the continuing need to increase and refine our basic understanding on the internal and morphological structures of plants. This is true in the case of *L. philippinense*. As a vulnerable species, it needs thorough characterisation and documentation for baseline information for conservation measures (Volis 2015).

Our description of *Lilium philippinense* is consistent with the original description by Baker. However, we are able to describe the fruits and seeds that were not done in its original publication. Also, Baker noted that individuals having two flowers are rare, but we found several individuals having two and few individuals having three. Our results affirm the pure white colour of taxon which contradicts the descriptions given by Napaldet (2017) and which clearly shows that specimen he described is *L. formosanum* and not *L. philippinense*.

Our result on variability of floral measurements in *L. philippinense*, in a way, contradicts the generally accepted observations that reproductive characters of plants were generally fixed. This observation is generally true in case of morphological descriptions of the taxon but is not applicable on the floral measurements particularly in terms of the lengths.

The mesophyll structure of *L. philippinense* is quite different from the common monocot leaves which have generally unificial mesophyll (Conklin *et al*. 2018). The palisade layer resembles a brick-like layer of rectangular cells, not elongated, which make it distinct from the spongy layer that is composed of generally larger cuboidal cells with larger air spaces. This finding supports the previous reports that mesophyll of genus *Lilium* is bifacial with a single palisade layer (Yembaturova & Korchagina 2011). Same bifacial mesophyll was also documented in *L. formosanum* (Napaldet 2017) and *L. candidum* (Özen *et al*. 2012) but no lobed or branching palisade cells were observed.

Anomocytic type was also reported in other species of *Lilium* and in families Boraginaceae, Ranunculaceae and Geraniaceae. The stomata are mostly concentrated in the abaxial epidermis. Few are noted sporadically in adaxial epidermis but only in the lamina base of middle and basal leaves. This contradicts the general notion that monocot have uniform distribution of stomata in the abaxial and adaxial epidermis. This also contradicts the findings of Zarinkamar (2006) that there are equal or more stomata in adaxial epidermis than abaxial in other species of Liliaceae such as *Allium atroviolaceum*, *Allium rubellum*, *Asparagus officinalis*, *Asphodeline dendroides* and others. However, these species are no longer under Liliaceae but were transferred to other related families under same order Liliales.

On the other hand, the cross sections of the mature and old stem of *L. philippinense* are similar with those of *L. candidum* described by Özen *et al*. (2012), with *L. polyphyllum* by Dhyani *et al*. (2009), and with *L. formosanum* by Napaldet (2017). The cortical fibres in the *Lilium* stem are schleranchymatic cells formed by secondary wall development that help plant organs for bending, folding and give support for weight and pressure (Fahn 1990; Vardar 1982; Yentür 1995).

In general, the anatomy of *L. philippinense* is a cross between a typical monocot and a typical dicot anatomy. It has a bifacial leaf structure (a common dicot character) but its spongy layer is not as widely spaced like in dicot leaf. The stem has a distinct cortex and pith (a dicot character) but has a scattered vascular bundle (a monocot character). Lastly, some roots have a narrow pith at the centre of the stele (a monocot character) while other roots have metaxylem elements at the centremost part (a dicot character). Further studies need to be conducted to determine the ecological significance of these features.

### Mathematics in *Lilium philippinense*

The mathematical precision in plants was long been recognised. Golden in 1892 summarised the application of mathematics in botany during that period, noting the tendency of science towards reducing results and conclusions into exactness. In physiological botany, several works have been conducted on reducing the principles of physiological phenomena into physics and chemistry thereby presenting them into mathematical formulae. During that period, works include gas absorption in plants, statistical similarity in successive generation, cell division in growing plants, correlation of moisture and blight in pear fruits, and many others. After these, many other works in this field have been performed and many others are still being conducted. In similar manner, much works on the mathematical application in plant ecology are progressing. These include the development of several biodiversity indices, plant distribution pattern, allometric models and many others.

However, mathematical works in plant morphology were not as robust as the other field of botany. Prominent works in this field include the discovery of Fibonacci sequence and, more recently, the tree architectural models by Halle (1974) and study of Nyman and Brown (1996) on the mathematical models that describe the growth and development of the giant kelp *Macrocystis pyrifera* in New Zealand. Our study would be an addition to these and could be the first report on the mathematical model on how vegetative traits (specifically plant height and stem diameter) dictate floral characters in plants as no other studies were cited in available literatures.

## Figures and Tables

**Table 1:** Morphometrics of *L. philippinense* Baker in the Cordillera Central Range.

Parameters	Mean	Minimum	Maximum	S.D.
Plant height (cm)	66.84	28.00	101.50	± 17.82
Stem diameter (cm)	0.47	0.30	0.80	± 0.12
Leaf length (cm)	13.08	7.50	18.00	± 2.50
Leaf width (cm)*	0.45	0.30	0.70	± 0.09
Number of flower per plant	1.08	1.00	3.00	± 0.34
Outer tepal length (cm)	19.89	13.50	24.50	± 2.20
Outer tepal width (cm)*	3.43	2.10	4.90	±0.66
Inner tepal length (cm)	19.67	12.90	23.80	± 2.33
Inner tepal width (cm)*	4.92	3.45	7.20	± 0.81
Anther length (cm)	0.83	0.50	1.80	± 0.24
Anther width (cm)	0.27	0.13	0.55	± 0.09
Filament length (cm)	15.09	9.30	18.50	± 1.62
Stigma width (cm)	0.76	0.50	1.20	± 0.14
Style length (cm)	14.08	9.00	17.00	± 1.64
Ovary length (cm)	3.56	2.00	4.70	± 0.65
Ovary diameter (cm)	0.42	0.30	0.60	± 0.06
Fruit length (cm)	5.19	3.90	6.10	± 0.75
Fruit diameter (cm)	1.67	1.30	2.20	± 0.30
Seed length (cm)	0.49	0.40	0.60	± 0.06
Seed width (cm)	0.75	0.55	0.85	± 0.08

*Notes*:

* = measured at the widest portion.

S. D. = Standard Deviation

**Table 2:** Morphometric difference between populations of *L. philippinense* Baker in the Cordillera Central Range.

Parameters	Atok 1	Atok 2	Atok 3	Atok 4	Atok 5	Buguias 1	Buguias 2	Kabayan 1	Kabayan 2	Bokod 1
Plant height (cm)	77.5^bc^	71.44^bc^	58.80^ab^	60.60^b^	81.30^bc^	36.10^a^	69.00^bc^	92.50^c^	64.30^b^	68.60^bc^
Stem diameter (cm)	0.45^a^	0.52^a^	0.48^a^	0.41^a^	0.58^a^	0.44^a^	0.52^a^	0.46^a^	0.40^a^	0.35^a^
Leaf length (cm)	11.96^ab^	14.92^bc^	14.04^bc^	13.04^bc^	13.76^bc^	8.18^a^	10.72^ab^	15.04^c^	13.24^bc^	12.70^bc^
Leaf width (cm)*	0.38^a^	0.47^ab^	0.47^ab^	0.43^ab^	0.55^b^	0.35^a^	0.40^a^	0.41^ab^	0.42^ab^	0.43^ab^
Outer tepal length (cm)	19.96^bc^	20.64^bc^	19.92^bc^	18.08^ab^	20.28^bc^	16.00^a^	21.90^c^	21.84^c^	20.60^bc^	19.58^abc^
Outer tepal width (cm)*	3.32^ab^	3.68^b^	3.94^b^	3.36^ab^	4.10^b^	2.56^a^	3.57^ab^	4.08^b^	3.06^ab^	3.04^ab^
Inner tepal length (cm)	20.22^bc^	20.04^bc^	20.00^bc^	17.58^ab^	19.80^bc^	15.18^a^	21.62^c^	21.38^bc^	20.58^bc^	20.46^bc^
Inner tepal width (cm)*	4.56^abc^	5.28^abc^	5.82^c^	4.70^abc^	5.78^bc^	4.09^a^	5.23^abc^	5.08^abc^	4.62^abc^	4.36^ab^
Anther length (cm)	0.90^a^	0.86^a^	1.08^a^	0.78^a^	1.09^a^	0.92^a^	0.97^a^	0.82^a^	0.72^a^	0.82^a^
Anther width (cm)	0.33^ab^	0.28^ab^	0.40^b^	0.27^ab^	0.34^ab^	0.26^ab^	0.30^ab^	0.26^ab^	0.23^a^	0.30^ab^
Filament length (cm)	15.34^ab^	14.90^ab^	14.72^ab^	13.24^ab^	14.58^ab^	12.94^a^	15.90^ab^	15.88^ab^	15.52^ab^	16.30^b^
Stigma width (cm)	0.60^a^	0.72^abc^	0.80^abc^	0.70^abc^	0.95^c^	0.68^ab^	0.72^abc^	0.80^abc^	0.78^abc^	0.90^bc^
Style length (cm)	14.76^ab^	13.86^ab^	13.62^ab^	12.80^ab^	13.94^ab^	11.86^a^	15.78^b^	15.34^b^	14.24^ab^	14.65^ab^
Ovary length (cm)	3.80^a^	4.02^a^	3.74^a^	3.16^a^	3.92^a^	2.74^a^	3.54^a^	3.80^a^	3.44^a^	3.35^a^
Ovary diameter (cm)	0.43^a^	0.45^a^	0.46^a^	0.39^a^	0.46^a^	0.35^a^	0.45^a^	0.43^a^	0.38^a^	0.35^a^

*Note*:

* = measured at the widest portion, means with the same letter in a row are not significantly different at 0.05 Tukey’s Test

**Table 3:** Correlation analyses of plant height and stem diameter with vegetative and reproductive morphometrics.

Plant characters	Plant height	Stem diameter
Leaf length	0.662**	0.319*
Leaf width	0.283	0.412**
Number of flowers	0.272	0.625**
Outer tepal length	0.649**	0.291
Outer tepal width	0.599**	0.462**
Inner tepal length	0.663**	0.283
Inner tepal width	0.367*	0.457**
Anther length	0.112	0.526**
Anther width	0.086	0.079
Filament length	0.400*	0.114
Stigma width	0.331*	0.365*
Style length	0.559**	0.278
Ovary length	0.492**	0.466**
Ovary diameter	0.396*	0.451**

*Note*:

* significant correlation at 0.05 Pearson Correlation;

** highly significant correlation at 0.01 Pearson Correlation

**Table 4:** Regression models using plant height and stem diameter to predict some vegetative and reproductive morphometrics.

Plant characters	Regression Model	*r*^2^ value
Leaf length	= 1.4437 × PH^0.5143^	0.4998
Number of flowers per individual	= 7.0513(SD^2^) − 5.1151(PH) + 1.9128	0.4444
Outer tepal length	= 0.6243 × PH^0.4128^	0.4137
Outer tepal width	= 5.9 × PH^0.2879^	0.4633
Inner tepal length	= −0.0014(PH^2^) + 0.2917(PH) + 7.149	0.4976
Inner tepal width	= 3.332 (SD^2^) + 0.8495(SD) + 3.8371	0.3871
Style length	= 0.0009(PH^2^) + 0.1625(PH) + 7.0969	0.3575

*Note*: PH = plant height; SD = stem diameter

**Table 5:** Biometric measurement of *L. formosanum* in the Cordillera Central Range.

Part of plant/parameter		Minimum	Maximum	Mean	S. D.
Bulb	Length (cm)	1.80	4.00	2.78	±0.75
	Width (cm)	2.00	4.50	3.10	±0.96
Stem	Length (cm)	39.60	190.00	87.77	±39.90
	Diameter (cm)	0.30	1.00	0.51	±0.20
Leaves	Length (cm)	6.00	24.50	14.10	±4.84
	Width (cm)	0.30	1.10	0.62	±0.20
Pedicel	Length (cm)	3.00	16.50	8.64	±4.25
Outer tepal	Length (cm)	13.50	17.00	15.13	±0.78
	Width (cm)*	2.00	3.10	2.63	±0.34
Inner tepal	Length (cm)	13.40	16.70	14.84	±0.74
	Width (cm)*	3.40	4.90	4.14	±0.39
Anther	Length (cm)	0.50	1.50	0.80	±0.33
Filament	Length (cm)	10.50	12.10	11.37	±0.45
Ovary	Length (cm)	3.80	7.00	4.59	±0.71
	Width (cm)	0.30	0.50	0.38	±0.06
	Style Length(cm)	7.30	8.90	8.05	±0.34
Stigma	Diameter (cm)	0.70	1.40	0.92	±0.20
Capsule (Fruit)	Length (cm)	4.00	10.10	7.22	±1.60
	Diameter (cm)	1.80	2.50	2.14	±0.21
Seed	Length (cm)	0.50	0.90	0.75	±0.09
	Width(cm)	0.50	0.50	0.46	±0.06

## Data Availability

The data that support the findings of this study are available from the corresponding author, upon reasonable request.
